# Docosatetraenoyl LPA is elevated in exhaled breath condensate in idiopathic pulmonary fibrosis

**DOI:** 10.1186/1471-2466-14-5

**Published:** 2014-01-27

**Authors:** Sydney B Montesi, Susan K Mathai, Laura N Brenner, Irina A Gorshkova, Evgeny V Berdyshev, Andrew M Tager, Barry S Shea

**Affiliations:** 1 Pulmonary and Critical Care Unit, Massachusetts General Hospital and Harvard Medical School, Boston, MA, USA; 2 Center for Immunology and Inflammatory Diseases, Massachusetts General Hospital and Harvard Medical School, Boston, MA, USA; 3 Division of Pulmonary Sciences and Critical Care Medicine, University of Colorado Denver, Aurora, CO, USA; 4 Department of Medicine, University of Illinois at Chicago, Chicago, IL, USA

**Keywords:** Idiopathic pulmonary fibrosis, Exhaled breath condensate, Lysophosphatidic acid

## Abstract

**Background:**

Idiopathic pulmonary fibrosis (IPF) is a progressive and fatal disease with no effective medical therapies. Recent research has focused on identifying the biological processes essential to the development and progression of fibrosis, and on the mediators driving these processes. Lysophosphatidic acid (LPA), a biologically active lysophospholipid, is one such mediator. LPA has been found to be elevated in bronchoalveolar lavage (BAL) fluid of IPF patients, and through interaction with its cell surface receptors, it has been shown to drive multiple biological processes implicated in the development of IPF. Accordingly, the first clinical trial of an LPA receptor antagonist in IPF has recently been initiated. In addition to being a therapeutic target, LPA also has potential to be a biomarker for IPF. There is increasing interest in exhaled breath condensate (EBC) analysis as a non-invasive method for biomarker detection in lung diseases, but to what extent LPA is present in EBC is not known.

**Methods:**

In this study, we used liquid chromatography-tandem mass spectrometry (LC-MS/MS) to assess for the presence of LPA in the EBC and plasma from 11 IPF subjects and 11 controls.

**Results:**

A total of 9 different LPA species were detectable in EBC. Of these, docosatetraenoyl (22:4) LPA was significantly elevated in the EBC of IPF subjects when compared to controls (9.18 pM vs. 0.34 pM; p = 0.001). A total of 13 different LPA species were detectable in the plasma, but in contrast to the EBC, there were no statistically significant differences in plasma LPA species between IPF subjects and controls.

**Conclusions:**

These results demonstrate that multiple LPA species are detectable in EBC, and that 22:4 LPA levels are elevated in the EBC of IPF patients. Further research is needed to determine the significance of this elevation of 22:4 LPA in IPF EBC, as well as its potential to serve as a biomarker for disease severity and/or progression.

## Background

Idiopathic pulmonary fibrosis is a progressive and ultimately fatal disease in which normal lung is replaced by fibrous scar tissue. The cause of the disease is unknown; however, exposure to refluxed gastric acid, occupational exposures, and viral infections have been postulated as inciting insults [1-3]. The average duration from diagnosis to time of death is 2–3 years [4]. Diagnosis is made either by pathology consistent with usual interstitial pneumonia or radiographic findings showing areas of fibrosis and honeycombing in the absence of an alternate diagnosis [5]. Once the diagnosis of IPF is made limited options exist for treatment except for lung transplantation.

Recent advances have occurred in our understanding of the mechanisms involved in IPF pathogenesis. Specifically, aberrant wound healing responses to tissue injury, such as epithelial cell apoptosis, increased vascular permeability, extravascular coagulation, and fibroblast migration and activation, have all been implicated in the development of lung fibrosis [6,7]. Research efforts have focused on identifying molecular pathways central to the progression from normal to fibrotic lung, as a better understanding of such pathways may provide potential targets for pharmacologic therapy and biomarkers to aid in diagnosis or prognosis [7]. One such area of interest involves the role of lysophosphatidic acid (LPA) in the development and progression of pulmonary fibrosis.

LPA is a biologically active lysophospholipid that has been shown to mediate numerous biological processes thought to contribute to tissue fibrosis [7]. Structurally, LPA consists of glycerol-phosphate with a single fatty acid esterified at the *sn-*1 or *sn*-2 position. There are numerous LPA species present in biological fluids, identified by the length and degree of saturation of the fatty acid moiety [8]. The majority of extracellular LPA is produced from lysophosphatidylcholine (LPC) by the enzyme autotaxin (also known as lysophospholipase D) [9,10]. LPA’s activity is mediated by interaction with specific G protein-coupled receptors, six of which have been definitively identified (LPA_1-6_) [7,11,12]. The role of LPA and its receptors has been investigated in the development of fibrosis in multiple organ systems, including the lung, liver, kidneys, skin and peritoneum [13-17]. In the setting of lung injury, LPA has been shown to contribute to epithelial cell death, increased vascular permeability, and fibroblast migration and persistence via interaction with the LPA_1_ receptor, and genetic deficiency or pharmacologic inhibition of LPA_1_ confers protection against bleomycin-induced lung fibrosis in mice [13,18,19]. Furthermore, LPA is elevated in the BAL fluid of IPF patients and contributes to fibroblast migration into the injured airspaces in this disease [13]. Based on the apparent importance of the LPA-LPA_1_ pathway for the development of lung fibrosis, a Phase II clinical trial of an oral LPA_1_ antagonist for the treatment of IPF has recently been initiated (ClinicalTrials.gov identifier: NCT01766817). Recent evidence indicates that the LPA_2_ receptor can also mediate profibrotic effects of LPA, such as activation of latent transforming growth factor-β (TGF-β), and genetic deficiency of this receptor also results in protection against the development of lung fibrosis in mice [13,20,21].

Given its potentially important and central role in the development of pulmonary fibrosis, LPA is not only a therapeutic target but also a potential biomarker in IPF. While elevated LPA levels have been detected in the BAL from IPF patients [13], the extent to which LPA is present and detectable in exhaled breath condensate (EBC) is not known. EBC has become an area of interest for potential biomarker analysis in respiratory diseases [22]. Collection of EBC can be performed in a low-cost and non-invasive manner. For the detection of certain biologic molecules, correlation has been demonstrated between EBC and BAL results, though further research is needed [23]. In addition to volatile gases, EBC contains nonvolatile particles representing airway and alveolar lining fluid contents [24]. The ability to analyze components from the lining of the respiratory epithelium offers great potential for biomarker discover. EBC has been studied in different respiratory diseases, including asthma and COPD [25,26]. However, few studies have analyzed EBC in the setting of interstitial lung disease, specifically IPF [27,28]. If LPA were detectable in EBC, it may provide information about the disease and/or the disease course. In this study we sought to assess for the presence of LPA in plasma and EBC and determine if differences exist in the amount of LPA in subjects with IPF versus controls.

## Methods

### Study subjects

Subjects with IPF were identified from those being cared for in the Massachusetts General Hospital (MGH) outpatient pulmonary clinic or inpatient pulmonary consult service. For inclusion in this study, subjects had to meet criteria for a diagnosis of IPF based on the recent joint consensus statement of the American Thoracic Society (ATS), European Respiratory Society (ERS), Japanese Respiratory Society (JRS), and Latin American Thoracic Association (ALAT) [5]. Controls were recruited through the Partners Healthcare System Research Study Volunteer Program (RSVP). Controls were non-smoking individuals at least 50 years of age without a history of chronic lung disease. Study approval was obtained through the Partners Institutional Review Board, and informed consent was obtained on all subjects. Eleven IPF subjects and eleven controls were included in this study. EBC was obtained on all subjects, and plasma was obtained on all 11 IPF patients and 10 of the controls.

### Exhaled breath condensate (EBC) collection

EBC was collected using the handheld RTube™ exhaled breath condensate collector (Respiratory Research, Inc.), according to the manufacturer’s instructions, and following the ATS/ERS methodological recommendations for EBC collection [29]. Collection was performed during 10 minutes of tidal breathing, with a nose clip in place, using a cooling chamber pre-cooled to -20°C. EBC samples were placed in aliquots and immediately frozen and stored at -80°C until analysis.

### Plasma collection

Blood was obtained via venipuncture into tubes containing CTAD (citrate-theophylline-adenosine-dipyridamole) additive, in order to potently inhibit platelet activation, as activated platelets are known to release abundant amounts of LPA [30]. Within 30 minutes of collection, whole blood was centrifuged at 1500 × g for 15 minutes to obtain plasma, which was then placed in aliquots and immediately frozen and stored at -80°C until analysis.

### Lipid extraction

EBC samples were subjected to lipid extraction using the modified Bligh and Dyer method as described [31,32]. Briefly, lipid extraction was initiated by adding 2 ml methanol and 1 ml chloroform to 0.5 ml EBC, followed by the addition of 2 pmol C17-LPA (internal standard; Avanti Polar Lipids, Alabaster, AL, USA). Extraction was allowed for 30 minutes with the samples kept on ice. Then, phase separation was achieved by adding 1 ml chloroform and 1.3 ml 0.1 N HCl with vigorous vortexing. The chloroform phase was collected, the solvent was evaporated under a stream of nitrogen gas, and residues were dissolved in methanol and transferred into autosampler vials for LC-MS/MS analysis.

### Measurement of LPA species by liquid chromatography-tandem mass spectrometry (LC-MS/MS)

LPA levels were determined using electrospray ionization liquid chromatography tandem mass spectrometry (ESI-LC/MS/MS) with an AB Sciex 5500 QTRAP hybrid triple quadrupole/ion trap mass spectrometer coupled with an Agilent 1200 liquid chromatography system. Lipids were separated on Ascentis Express C8 (75 × 2.1 mm, 2.7 um) column using methanol:water:HCOOH, 60:40:0.5, v/v with 5 mM NH_4_COOH as solvent A and acetonitrile:chloroform:water:HCOOH, 80:20:0.5:0.5, v/v with 5 mM NH_4_COOH as solvent B. LPA molecular species were analyzed in negative ionization mode with declustering potential and collision energy optimized for each LPA molecular species. Individual saturated and unsaturated LPA molecular species (16:0-, 17:0-, 18:0-, 18:1-, and 20:4-LPA, all obtained from Avanti Polar Lipids, Inc., Alabaster, AL) were used as reference compounds. 17:0-LPA was used as the internal standard, and LPA quantitation was performed by creating standard curves with variable amounts of each available LPA molecular species versus fixed amount of the internal standard (17:0-LPA). Total lipid extract from fetal bovine serum was used as a source of otherwise unavailable LPA molecular species to determine their chromatographic behavior and parameters of ionization and collision-induced decomposition, and the quantitation of these LPA molecular species was achieved via the use of the best possible approximation from the standard curves obtained with available individual LPA standards. The identification of LPA molecular species was achieved via monitoring for selected transitions from molecular to product (m/z 153) ions specific for each LPA molecular species, and by the analyte retention time identified by the available LPA standards and by comparing with LPA extracted from bovine serum.

### Statistical analyses

Statistical analysis was performed using Prism 6.0 (GraphPad Software, Inc.). Differences in LPA levels between IPF patients and controls were analyzed for statistical significance using a two-tailed Student’s t-tests or Mann Whitney tests for parametric and nonparametric data, respectively. To adjust for multiple comparisons, we used the Bonferroni method to calculate the accepted α (Type I) error rate for each individual comparison performed, keeping the family-wise error rate at 0.05. Therefore, for EBC LPA levels, in which 9 different LPA species measured were measured, p values ≤ 0.0055 (0.05/9) were considered statistically significant. For plasma LPA levels, in which 13 different LPA species were measured, p values ≤ 0.0038 (0.05/13) were considered statistically significant.

## Results

### Patient characteristics

Relevant demographic and clinical data for IPF subjects (n = 11) and controls without lung disease (n = 11) on whom EBC and plasma LPA measurements were performed are summarized in Table 1. Of the 11 IPF subjects, 6 were diagnosed by surgical lung biopsy, and 5 were diagnosed by clinical and radiographic criteria alone. The mean age was 67.7 (+/- 8.5) years in the IPF group and 68.2 (+/- 7.1) years in the control group. The male to female ratio was 10:1 in the IPF group and 9:2 in the control group. There were no current smokers in either group; however, both groups contained former smokers. Spirometry data were available on 8/11 subjects and DLCO (diffusion capacity of carbon monoxide) data on 7/11 IPF subjects as shown in Table 1. Spirometry was not obtained on control subjects. Of the available pulmonary function results, the majority of testing (5/8) was performed on the day of EBC and plasma collection. All pulmonary function testing was performed within 15 days of sample collection. Supplemental oxygen was needed for 7/11 of IPF subjects. None of the IPF subjects were taking inhaled corticosteroids at the time of data collection.

**Table 1:** Subject characteristics

	**IPF (n = 11)**	**Controls (n = 11)**
Age (yrs)	67.7 (+/- 8.5)	68.2 (+/- 7.1)
Sex (M/F)	10/1	9/2
Smoking (current/ever/never)	0/8/3	0/9/2
FVC (% predicted)	60.5 (+/- 15.1)	N/A
TLC (% predicted)	61.5 (+/- 8.1)	N/A
DLCO [Hb] (% predicted)	43.7 (+/- 20.8)	N/A

Data are presented as mean +/- SD as appropriate. N/A refers to data that was not available or collected.

### Levels of LPA in exhaled breath condensate and plasma

**Figure 1:**
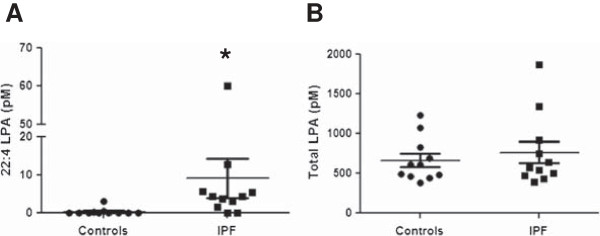
**Exhaled breath condensate (EBC) LPA levels.** Levels of docosatetraenoyl (22:4) LPA **(A)** and total LPA **(B)** in the exhaled breath condensate (EBC) of IPF patients and controls, as determined by liquid chromatography-tandem mass spectrometry (LC-MS/MS). Levels of 22:4 LPA, but not total LPA, were significantly higher in IPF patients vs. controls, with minimal overlap in EBC 22:4 LPA levels between the two groups. *p = 0.001 for the comparison of IPF vs. controls by Mann Whitney test.

Nine different LPA species were detected in the EBC from IPF subjects and controls (Table 2). Of these, docosatetraenoyl (22:4) LPA exhibited a statistically significant difference between the two groups, with levels being significantly higher in IPF patients compared to controls (9.18 vs. 0.34 pM; p = 0.001). Furthermore, there was minimal overlap between EBC 22:4 LPA levels in IPF patients and controls. It was detected at levels > 1.5 pM in 9/11 IPF patients but was undetectable in all but three of the controls, and in only one control was the level > 0.4 pM (Figure 1). For the remaining eight LPA species, no statistically significant differences were detected between the two groups; however, there were trends towards increased levels of 18:2 LPA and 20:3 LPA in EBC of IPF patients (p = 0.13 and p = 0.055, respectively). There was no significant difference in the total amount of LPA in EBC between the two groups (664.69 +/- 83.03 vs. 766.15 +/- 137.35 pM, p = 0.73). Thirteen different LPA species were detected in plasma from IPF subjects and controls (Table 3). None of these 13 species showed statistically significant differences between the two groups, nor was there a statistically significant difference in the total amount of LPA between the two groups (mean control LPA 77.90 +/- 22.31 nM and mean IPF 64.51 +/- 12.82 nM, p = 0.10).

**Table 2:** LPA levels in Exhaled Breath Condensate (EBC)

**LPA**	**IPF**	**Controls**	**p-value**
14:0	10.76 (2.54)	9.49 (1.79)	0.88
16:0	166.68 (33.74)	149.42 (20.53)	> 0.99
18:0	359.99 (101.57)	312.70 (62.14)	0.78
18:1	15.81 (9.06)	9.00 (3.38)	0.76
18:2	9.19 (1.47)	5.93 (1.03)	0.13
20:2	40.37 (1.96)	40.03 (1.64)	0.83
20:3	153.43 (6.74)	137.77 (3.90)	0.055
20:5	0.00 (N/A)	0.73 (0.53)	0.48
22:4	9.18 (5.19)	0.34 (0.27)	0.0010
Total	766.15 (137.35)	664.69 (83.03)	0.73

Data are reported as mean concentration in pM (standard error).

**Table 3:** LPA levels in plasma

**LPA**	**IPF**	**Controls**	**p-value**
14:0	0.11 (0.025)	0.19 (0.047)	0.11
16:0	6.05 (0.38)	6.93 (0.74)	0.29
16:1	0.66 (0.074)	0.97 (0.15)	0.071
18:0	3.69 (0.32)	3.46 (0.30)	0.60
18:1	5.10 (0.37)	6.05 (0.82)	0.29
18:2	19.40 (1.99)	24.56 (3.60)	0.21
20:2	0.54 (0.046)	0.56 (0.072)	0.74
20:3	3.13 (0.26)	3.64 (0.22)	0.16
20:4	11.89 (1.14)	13.96 (1.06)	0.20
20:5	1.55 (0.15)	2.31 (0.38)	0.051
22:4	0.92 (0.73)	0.88 (0.092)	0.70
22:5	2.98 (0.18)	3.10 (0.19)	0.67
22:6	10.06 (0.57)	11.29 (1.32)	0.39
Total	64.51 (3.87)	77.90 (7.05)	0.10

Data are reported as mean concentration in nM (standard error).

### Docosatetraenoyl (22:4) LPA and subject characteristics

The average EBC 22:4 LPA level in IPF patients was 9.18 +/- 5.19 pM. There was no correlation between 22:4 LPA levels and disease severity, as determined by percent predicted FVC or DLCO (data not shown). One subject had an EBC 22:4 LPA level of 60 pM, which far exceeded the standard deviation of the mean. This subject was a 46 year-old man with biopsy-proven usual interstitial pneumonia (UIP) who required hospital admission for worsening respiratory status in the setting of a suspected IPF exacerbation. Spirometry performed during the patient’s hospitalization and within 10 days of EBC collection demonstrated a severe restrictive deficit, with a FVC of 33% predicted. He subsequently developed respiratory failure and eventually underwent lung transplantation within one month of sample collection. Pathologic review of the explanted lung revealed UIP in the accelerated phase. None of the other ten IPF patients in this study were in the midst of IPF exacerbations during sample collection.

## Discussion

LPA has emerged as an important pro-fibrotic mediator in multiple organ systems, particularly the lungs, and the first clinical trial of an LPA receptor antagonist has recently been initiated in IPF patients (ClinicalTrials.gov identifier: NCT01766817). In this study, we analyzed the exhaled breath condensate (EBC) and plasma from eleven IPF patients and eleven controls without lung disease for the presence of lysophosphatidic acid (LPA), using liquid chromatography-tandem mass spectrometry (LC-MS/MS). We demonstrated that at least nine LPA species are detectable in EBC, and that one of these species, docosatetraenoyl (22:4) LPA, is significantly elevated in the EBC of IPF patients compared to controls. Thirteen LPA species were detectable in plasma; however, none of these differed significantly between the two groups.

Multiple species of LPA exist in biological fluids and are identified according to the composition of their fatty acid side chain. While all LPA species are thought to signal through LPA receptors, there are data indicating that the different species may have differing affinities for the various receptors [33]. Very little is known about 22:4 LPA specifically, and it is unclear whether or not its signaling profile differs significantly from that of other LPA species. Notably, unsaturated LPA species appear to have higher affinity for most LPA receptors than do saturated species [34]. In particular, long chain, polyunsaturated LPA species (like 22:4 LPA) have been shown to be the most potent activators of certain biological processes, such as platelet activation [35]. Therefore, it is possible that 22:4 LPA may have more potent pro-fibrotic effects compared to other LPA species, and that the increase in 22:4 LPA in the EBC of IPF patients may be playing a role in driving the disease process. It should be noted, however, that the amount of 22:4 LPA in EBC was only a small fraction of total LPA, which may argue against a significant pathophysiological role for this particular LPA species in IPF. The increase in 22:4 LPA may instead indicate the generation of LPA from a specific a specific source, such as lung epithelial cells, which are known to contain high levels of polyunsaturated phospholipids [36].

In addition to being a therapeutic target, LPA may also serve as a useful biomarker for IPF. Elevations in LPA have been detected in the bronchoalveolar lavage (BAL) fluid from mice after intratracheal bleomycin administration and from humans with known IPF [13]. 22:4 LPA was not specifically measured in this previous report of IPF patients, but it is detectable in BAL fluid, and it and other long-chain, polyunsaturated LPA species have been found to be elevated in BAL fluid in a mouse model of asthma and in human allergic airway inflammation [37,38]. Our data suggest that EBC 22:4 LPA levels may be a useful biomarker for IPF diagnosis and/or prognosis. From a diagnostic standpoint, our data demonstrate minimal overlap between EBC 22:4 LPA levels in IPF patients and controls. To be of true value in the diagnosis of IPF, EBC 22:4 LPA levels would have to be able to differentiate between IPF and other forms of chronic interstitial lung diseases, most notably nonspecific interstitial pneumonia (NSIP) and chronic hypersensitivity pneumonitis (HP). As such comparisons were not performed in this study, further research would be needed to fully evaluate the potential role of EBC 22:4 LPA levels as a diagnostic biomarker in IPF.

It is notable that the EBC 22:4 LPA level in one patient was far outside the standard deviation of the mean, and that this patient was in the midst of an IPF exacerbation at the time of sample collection. This observation raises the hypothesis that EBC 22:4 LPA levels may be a useful biomarker of disease activity and/or acute exacerbations in IPF. Analysis of our data failed to reveal an association between EBC 22:4 LPA levels and disease severity or outcomes (decline in pulmonary function or mortality), although this study was likely underpowered to detect any such associations. Further study of EBC LPA levels in IPF, specifically in patients with rapidly progressive disease and those suffering from acute exacerbations, may shed light on the potential role of EBC 22:4 LPA levels as a prognostic biomarker in this disease.

While BAL has long been considered the optimal means of sampling the alveolar surfaces for analysis, it is invasive and not without risk, especially in subgroups of patients with advanced respiratory disease, such as those with pulmonary fibrosis. In comparison, EBC provides a method for non-invasive sampling of the lower respiratory tract. There are concerns regarding the accuracy with which EBC reflects the distal lung microenvironment, however, as there is risk of contamination with oral and gastrointestinal secretions, as well as an unknown (and potentially variable) dilution factor due to condensed water vapor [24]. Recommendations regarding optimized EBC collection have been made to minimize contamination and variations in solute dilution [24,29]. In our current study, it is reassuring that the two most abundant LPA species detected in EBC (16:0 and 18:0) were also the two most abundant species measured in BAL fluid from control subjects in the IPF (unpublished data) and asthma studies referenced above, suggesting that our EBC samples accurately reflect the distal lung compartments [38]. Furthermore, the total LPA levels in our EBC samples are similar to those seen in BAL fluid, with respect to both the mean values and the standard deviations, suggesting that the dilution factors (and the variability thereof) may be similar for these two sample types [37].

Additional limitations to this study exist. Most notably, the sample size of the study was small. Though we were able to detect a difference in the amount of 22:4 LPA in IPF subjects versus controls, we were likely underpowered to detect differences in the other LPA species should any exist. While the subjects in the study population of interest all met current consensus guidelines for diagnosis of IPF, they differed in disease severity. Whether or not significant differences in LPA species could be detected when stratified for disease severity is not known. However, despite our limitations, we were able to detect a significant elevation in the amount of 22:4 LPA in EBC from IPF subjects. This study builds on previous work showing that LPA is increased in BAL fluid in IPF patients [13], and advances the current field of pulmonary research by showing that LPA can be extracted from EBC. Additional research is needed to determine any relationships between LPA species detectable in EBC and disease severity or progression in IPF.

## Conclusions

LPA is detectable in exhaled breath condensate (EBC), and 22:4 LPA levels are elevated in the EBC of IPF patients compared to controls.

## Abbreviations

IPF: Idiopathic pulmonary fibrosis; LPA: Lysophosphatidic acid; EBC: Exhaled breath condensate; BAL: Bronchoalveolar lavage; LC-MS/MS: Liquid chromatography-tandem mass spectrometry; FVC: Forced vital capacity; DLCO: Diffusion capacity of carbon monoxide.

## Competing interests

AMT has applied for the following patent: U.S. Patent Application, 12/450,051, filed September 9, 2009, “Lysophosphatidic Acid Receptor Targeting for the Treatment of Lung Disease”.

## Authors’ contributions

SBM assisted in data collection, performed statistical analyses, and prepared the draft of the manuscript. SKM and LNB assisted in subject enrollment and performed clinical data collection and analyses. IG and EVB performed lipid extraction and measurement of LPA species by LC-MS/MS. BSS and AMT designed and oversaw all aspects of this study. All authors read and approved of the final manuscript.

## Pre-publication history

The pre-publication history for this paper can be accessed here:

http://www.biomedcentral.com/1471-2466/14/5/prepub
